# Basic science in integrated curricula: A medical student experience

**DOI:** 10.1007/s40037-016-0290-4

**Published:** 2016-08-04

**Authors:** Milani Sivapragasam

**Affiliations:** Faculty of Medicine, McGill University, Montreal, Quebec Canada

Recent papers by Lisk and Sibbald have again raised the role of basic sciences in medical training – a debate stretching back to the original Flexner Report [1, 2]. For medical students like myself, Flexner’s vision for scientific rigor in medical education continues to resonate deeply. Many of us are trained in basic sciences prior to entering medicine. We arrive keen to learn basic science is no longer just the object of our fascination but the foundation of clinical medicine, a powerful tool used to care for human beings. But as I experienced first-hand during my own training, medical curricula often challenge these notions as impractical. As professors would say ‘you don’t need to know that’ or ‘I don’t use that in my clinical practice’, students felt discouraged in their attempts to understand the biological basis of medicine. Students’ intellectual curiosity eroded as we found ourselves memorizing lists of clinical features and drugs – so-called ‘practical clinical knowledge’ – while struggling to appreciate mechanistic underpinnings of the same diseases and treatments. Thus issues raised by Lisk and Sibbald are not abstract but have significant impact on identity construction in trainees. This experience spurred my involvement in curriculum reform to join the century-long effort to successfully integrate basic science in medical education.

My institution’s pre-clinical basic science training was recently restructured as an integration strategy: (1) the volume of content was dramatically reduced to teach more clinically relevant concepts; (2) basic science became taught in proximity to clinical science. Consistent with experimental findings [1, 3], this increased proximity on its own proved insufficient to integrate learning. Recognizing the issue lied in the nuances of teaching, we have been refining the curriculum to purposefully expose explicit connections between clinical and basic science in a manner that facilitates cognitive integration and conceptual coherence in learners [1, 3]. For example, in the musculoskeletal system, anatomists went beyond teaching muscle identification, function, innervation, etc. to include clinical case discussions, introduce the rationale for physical exam tests and partnered with radiologists to teach diagnostic skills – these approaches were well received by students who cited effectively using anatomy knowledge to reason through clinical presentations. Medical students see basic science as the language of medicine, a language that enables us to think and communicate. As medical curricula evolve in the next century, let us not lose sight of physician identity as rooted in the deep integration of multiple basic sciences in thinking and patient care.
